# Effect of a Synbiotic Supplement on Fear Response and Memory Assessment of Broiler Chickens Subjected to Heat Stress

**DOI:** 10.3390/ani11020427

**Published:** 2021-02-07

**Authors:** Ahmed Mohammed, Manal Mahmoud, Raj Murugesan, Heng-wei Cheng

**Affiliations:** 1 Department of Animal Sciences, Purdue University, 915 West State Street, West Lafayette, IN 47907, USA; ahmed.abd_elhafez@vet.au.edu.eg; 2 Department of Animal and Poultry Behavior and Management, Faculty of Veterinary Medicine, Assiut University, Assiut 71526, Egypt; 3 Department of Animal Hygiene, Faculty of Veterinary Medicine, Assiut University, Assiut 71526, Egypt; manalmahmoud@vet.au.edu.eg; 4 BIOMIN America Inc., Overland Park, KS 66210, USA; raj.murugesan@biomin.net; 5 USDA Agricultural Research Service, 125 South Russell Street, West Lafayette, IN 47907, USA

**Keywords:** broilers, heat stress, gut microbiota, mental status, gut-brain axis, stress indicator

## Abstract

**Simple Summary:**

Heat stress is a serious environmental problem, challenging poultry health and welfare globally, especially during summer season. The breeding program of faster-growing broiler chickens affects their biological homeostasis, causing structural and functional damage to the brain, leading to mental disorders. Improvement in the gut microbiota with synbiotic dietary supplements has become a useful biotherapeutic method for treating various diseases, including neuroinflammation-induced mental illness and memory damage. Therefore, this study aimed to assess the effect of a dietary synbiotic supplement on fear response and memory assessment in heat-stressed broiler chickens. We used 360 1-day-old broiler chicks and assigned them to one of three dietary treatments: a regular diet mixed with a synbiotic containing a probiotic (*Enterococcus faecium*, *Pediococcus acidilactici*, *Bifidobacterium animalis*, and *Lactobacillus reuteri*) and a prebiotic (fructooligosaccharides) at 0, 0.5, and 1.0 g/kg. Object memory, touch, novel object, isolation, and tonic immobility tests were conducted at relative days of age. At 42 day, blood was collected for detecting corticosterone and tryptophan concentrations and examining heterophile/lymphocyte ratios. The data suggest that the synbiotic-reduced heat-stress responses and related emotional disorder may be mainly caused by increasing the activation of the serotonergic system via the microbiota–gut–brain axis.

**Abstract:**

The aim of this study was to evaluate the effect of a synbiotic containing a probiotic (*Enterococcus faecium*, *Pediococcus acidilactici*, *Bifidobacterium animalis*, and *Lactobacillus reuteri*) and a prebiotic (fructooligosaccharides) on fear response, memory assessment, and selected stress indicators in broilers subjected to heat stress. A total of 360 1-day-old Ross 708 chicks were evenly divided among three treatments: a basal diet mixed with a synbiotic at 0 (G-C), 0.5 (G-0.5X), and 1.0 (G-1.0X) g/kg. After 15 d, the broilers were exposed to 32 °C for 9 h daily until 42 d. The object memory test was conducted at 15 day; touch, novel object, and isolation tests were conducted at 35 day; tonic immobility (TI) took place at 41 day. At 42 day, plasma corticosterone and tryptophan concentrations and heterophile/lymphocyte (H/L) ratios were measured. Compared to controls, synbiotic-fed broilers, regardless of concentration, had a shorter latency to make the first vocalization, with higher vocalization rates during the isolation test (*p* = 0.001). the G-1.0 group had the lowest H/L ratio (*p* = 0.001), but higher plasma tryptophan concentrations and a greater number of birds could reach the observer during the touch test (*p* = 0.001 and 0.043, respectively). The current results indicate that the synbiotic can be used as a growth promoter to reduce the fear response and stress state of heat-stressed broilers.

## 1. Introduction

Heat stress (HS) is a detrimental environmental stressor affecting global broiler meat production. Particularly, climate change in recent decades has resulted in more hot days, with more intense and frequent unexpected heat waves [1]. Modern broiler chickens have been selected continuously for maximum growth rate and high feed conversion efficiency over 6–8 weeks [2], by which the breeding program affects broiler biological homeostasis, resulting in immature or impaired metabolism, immunity, and antioxidant status, as well as susceptibility to inflammation and infection. All these changes affect production performance negatively, accelerate mortality and morbidity, and consequently cause heavy economical losses [3,4]. Excessive mortality due to HS is commonly seen in commercial flocks of broilers. For those broilers which survive high temperatures, economically important production traits such as feed intake, body weight gain, meat quality, and feed efficiency are detrimentally affected. [5,6]. Heat-stress-associated annual economic loss in poultry in the United States alone is estimated at 128–240 million USD annually [7]. 

Numerous studies have revealed that HS can directly and indirectly affect humans’ and animals’ biological functions [4,8], causing brain structural and functional damage, leading to mental disorders [9,10]. The hypothalamus, as the neuroendocrine regularity center, is especially critical for thermoregulation. Previous studies have reported that HS activates the hypothalamic–pituitary–adrenal (HPA) axis to release corticosterone (CORT) in animals, including broilers [11,12]. Corticosterone has been used as a stress indicator and chronic stress-induced CORT secretion leads to mental illness and mood disorders in humans and experimental animals [13,14]. Summer anxiety and mood swings are often seen in humans suffering from high heat and humidity [9]. In addition, depressive- and anxiety-like behaviors are seen in CORT-induced depression in mice [14]. Similarly, hyperthermia causes both physiological and behavioral changes in chickens. Fearfulness, as one abnormal behavior, has been used as an indicator for evaluation of birds’ adaptability to physical, physiological, and psychological stressors [15]. Fearfulness tests including the novel object test, touch test, isolation test, and tonic immobility test, are regularly used in psychological investigations in humans and various animals, including poultry [16,17]. Some studies have revealed a relationship between fearful reactions and neuroendocrine pathways, including the adrenergic and serotonergic systems. Zulkifli et al. [18] reported that HS (34 + 1 °C for 3 h) increases circulating CORT and heat shock protein 70 concentrations in high-fear-response broilers, with enhanced tonic immobility reactions. 

Hyperthermia-induced memory loss and emotional damage may also take place through the disruption of the function of the microbiota–gut–brain axis [19]. Heat stress affects intestinal bacterial composition [20] and damages intestinal barrier integrity, increasing intestinal permeability, “leaky gut” [21,22], and consequently leading to elevated systemic lipopolysaccharide (LPS) levels [23]. Toxemia further causes neuroinflammation with neurological disorders and related emotional and mental damage [24,25].

Targeting the gut microbiota with fecal microbiota transplantation, prebiotics, probiotics, and synbiotics has become a useful biotherapeutic method for treating various diseases, including neuroinflammation-induced mental illness [26,27] and psychosocial disorders such as depression- and anxiety-like behaviors in humans and related animal models [28,29]. Synbiotics may be more efficient than prebiotics and probiotics, as synbiotics are a synergistic mixture of probiotics and prebiotics. Probiotics are live microorganisms that improve the survival and implantation of live beneficial microbes in the gut, either by metabolically activating or by the stimulation of beneficial bacteria [30], and prebiotics are nondigestible fiber compounds that have a useful influence on the host by selectively enhancing the survival and growth of healthy microbial species in the gut [31,32]. Several synbiotics [33,34,35] have been used as growth promoters and immune enhancers to increase production and health in broilers under both thermoneutral and hot ambient temperatures [36], while other synbiotics [37,38] had no effect on stress reactions. These contradictory effects could be due to the diversity and concentration of the synbiotics and/or experimental species used [39]. Furthermore, few studies have examined synbiotic effects on fear response and memory ability in broilers. Therefore, the objective of this study was to investigate the effect of a synbiotic supplement (a mixture of fructooligosaccharides and four selected microbial strains) on the fear response, memory ability and selected stress indicators in heat-stressed broiler chickens. We hypothesized that the dietary synbiotic supplementation would alleviate HS-negative effects on broiler health and welfare by preventing or diminishing stress-associated fear responses and memory damage.

## 2. Materials and Methods 

### 2.1. Ethical Approval

All experimental procedures and animal handling and care were approved by the Animal Care and Use Committee of Purdue University, protocol number: 1712001657, and the animals were housed in accordance with the guidelines of the Federation of Animal Science Societies at the Animal Research and Education Center of Purdue University (West Lafayette, IN, USA).

### 2.2. Synbiotic

The synbiotic was used in this study. It contains a prebiotic (fructooligosaccharides) and a probiotic mixture of four microbial strains selected from four different parts of the gastrointestinal track of chickens (*Enterococcus faecium* from Jejunum, *Pediococcus acidilactici* from cecum, *Bifidobacterium animalis* from ileum, and *Lactobacillus reuteri* from crop). It was confirmed to be safe for poultry by The European Food Safety Authority’s (EFSA) and it can be mixed with feed at a rate of 1 kg per ton.

### 2.3. Animals and Housing

Three-hundred-and-sixty 1-day-old male broiler chicks (Ross 708 strain; Pine Manor/Miller Poultry, Goshen, IN, USA) were weighed and allocated to 24-floor pens (110 × 110 cm per pen) of 15 birds each (*n* = 8 per treatment) with equal average body weight in a temperature-controlled room at the Poultry Research Farm of Purdue University. Broiler chickens were managed according to the guidelines of Aviagen [40], and wood shavings were used as flooring material. The chicks were raised at 34 °C for 1 day, then gradually decreased to 27 °C after 14 day. After 15 day, the chicks were stressed daily at 32 °C for 9 h (08:00–17:00 h), then returned to the regular room temperature during the evening and early morning hours until 42 day of age, the end of the experiment [41]. The room relative humidity was 55–60% during the experiment. Data loggers (HOBO^®^, Onset Computer Corporation, Bourne, MA, USA) were used to record the room temperature and humidity. The lighting program was gradually decreased from 23 light:1 dark (1:00–2:00 a.m.) at 30 lux up to the first 7 day of age, then 20 light:4 dark (1:00–5:00 a.m.) at 10 lux until 42 day of age.

### 2.4. Dietary Treatments

The 24 pens were randomly assigned to one of three dietary treatments: a regular mash diet mixed with the synbiotic at 0 (G-C), 0.5 (10^6^ CFU/g) (G-0.5), and 1.0 (2 × 10^6^ CFU/g) (G-1.0) g/kg feed. The synbiotic dietary treatments were made by the step-up procedure, as explained in detail by Mohammed et al. [41]. In brief, a small amount of the basal diet was mixed with the respective amount of the synbiotic in a small batch, and then this small batch was gradually integrated with a larger amount of the basal diet until the total amount was homogeneously mixed. The treatments were started at 1 day of age (Table 1).

### 2.5. Behavioral Tests

The following tests assessing the broiler chickens’ memory ability and fear response were conducted by observers in this study. To minimize the potential effects of circadian variations on the behavior as well as the concentration of neurohormones, the following tests were performed by repeating the cycle of G-C, G-0.5, and G-1.0 until the end of each test, and, to avoid using the same birds repeatedly for the following tests, the birds were marked with different color leg bands after each test. 

#### 2.5.1. Object Memory Test

**Figure 1:**
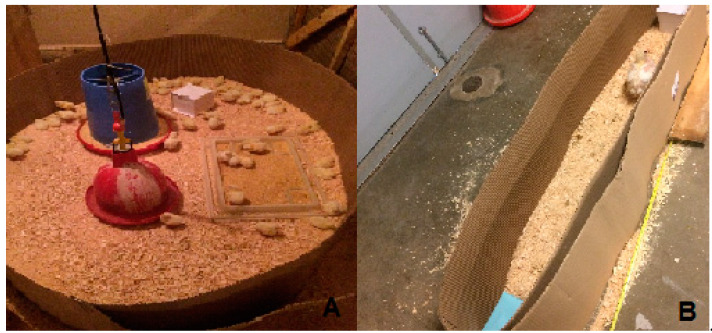
The examples of the object memory test. (**A**) During the imprinting phase from 1 to 7 day, a white block was placed inside each pen; and (**B**) during the test phase at 15 day, the tested bird was placed at the middle of the runaway board with the randomly placed imprinted object (white block) and a novel object (blue block) at one of its ends (16 birds per treatment).

A memory test was conducted after 15 day by following the protocol adapted from McCabe and Horn [42]. Briefly, during the imprinting phase from 1 to 7 day, all the chicks were reared with an imprinted object (a white block, 5 × 5 × 5 cm). The block was located near the feeder of each pen, in the same location. A 120 cm runaway board with sidewalls to block the visual stimulations was used for the test (Figure 1). It provided the tested chick with front or behind sight only, but the chick was able to change direction and run toward either end. During the test, in the afternoon, after 15 day (6 h after HS inducted, Acute HS), the imprinted object (the white block) and a novel object (a blue block with the same size and shape) were randomly placed on one end of the runaway board. Each of the tested chicks (two birds per pen and a total of 16 birds per treatment) was placed at the middle of the runaway board and the distance toward the imprinted object was measured. The data were presented as the mean of the distances traveled by the two birds.

#### 2.5.2. Novel Object Test

**Figure 2:**
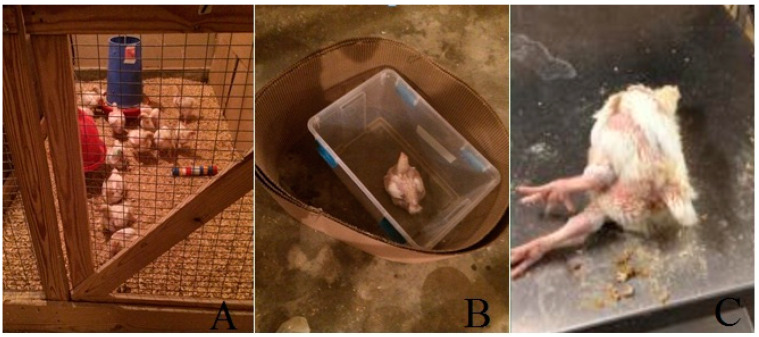
The examples of fear response tests. (**A**) Novel object test; (**B**) isolation test; and (**C**) tonic immobility test.

The test was performed by modified published method [16]. Briefly, in the morning of day 35 (HS for 21 days), each time, a plastic pipe (4 cm × 50 cm) painted with 5 different colors was allocated at each of the 3 locations (far end, center, and near entrance) per pen (Figure 2A). The observation of the birds’ fear response was conducted 2 min later by recording the number of birds within 30 cm distance from the novel objects. The pen data were presented as the mean of the bird number during the 3 observations.

#### 2.5.3. Touch Test

In the afternoon of day 35 (HS for 21 days), the touch test was carried out by following the published method [16]. Briefly, each time, an observer entered the pen and gently sat down at one of the three locations (far end, center, and near entrance), waited for 2 min, and then tried to touch the birds that were in reach. The mean of touched birds at the three locations was calculated per pen. To avoid contamination of the pen litter, the observer wore new protective boots for each pen. 

#### 2.5.4. Isolation Test

The isolation test was conducted by using an isolation box (55 cm length × 55 cm width × 85 cm height) in a separate room to avoid visual or auditory contact with conspecifics and the observer. In the evening of day 35 (HS for 21 days), each of the tested birds (two birds per pen and 16 birds per treatment) was placed in the middle of the isolation box for 2 min, and the time until the first vocalization, and the number of vocalizations during the test, were recorded (Figure 2B) [17]. 

#### 2.5.5. Tonic Immobility (TI)

A tonic immobility test was conducted following the previously published protocol [17]. Briefly, at 41 day (HS for 27 days), each of the tested birds (two birds per pen and 16 birds per treatment) was laid in a cradle upside down and held with slight pressure for 5 s to initiate a state of tonic immobility (Figure 2C). When pressure was removed, the duration of immobility was measured. If the bird righted itself in less than 10 s, the restraining procedure was repeated. The duration of TI was considered 0 s if TI was not induced after three attempts, while the birds were removed from the cradle after 600 s if no attempt to right themselves was made.

### 2.6. Blood sample Collection

At 42 day (HS for 28 days), two untested broilers per pen were randomly taken for blood collection (16 birds per treatment). Each bird was sedated with sodium pentobarbital (30 mg/mL, i.v.) and then 6 mL of blood was collected by cardiac puncture within 2 min after removal from its home pen. Duplicate blood smears were prepared by using a previously published routine laboratory method [43]. The blood samples were centrifuged at 3000× *g* for 15 min at 4 °C. The plasma was collected and kept at −80 °C until analyses.

#### 2.6.1. Heterophil/Lymphocyte (H/L) Ratio

Blood smears were dried at room temperature and then stained with Hema 3 Stain (Thermo Fisher Scientific Inc., Waltham, MA, USA) within 3 h after preparation. Two hundred white blood cells per bird were counted (one hundred cells per slide) under a light microscope at 2000× magnification. Lymphocytes and heterophils were distinguished based on their characteristics, as described by Campbell [44], then the H/L ratio was calculated [43].

#### 2.6.2. Plasma Tryptophan and Corticosterone Analyses 

Analyses of the plasma concentrations of tryptophan and CORT were performed by using the relative commercial chicken ELISA kits following the manufacturer’s instructions (MyBioSource, Inc., San Diego, CA, USA and Arbor Assays LLC, Ann Arbor, MI, USA, respectively).

### 2.7. Statistical Analysis

The experimental design was conducted in a completely randomized design. The overall effect of the synbiotic supplementation was analyzed by one-way analysis of variance (ANOVA, SAS Institute, Cary, NC, USA), with the pen considered as the experimental unit (n = 8). The synbiotic treatment was the fixed effect, and the two birds within a pen served as a subsample. The averaged mean of each parameter collected from the birds was presented for the statistical analysis, since its Coefficient Variation was less than 15%. The normality of the data was analyzed by the Shapiro–Wilk test. The data transformation was performed when variances were not homogeneous, and the untransformed results were presented due to the similarity of statistical trends between untransformed and transformed data [45]. The Tukey–Kramer test was used to test individual differences when a significant main effect was detected. Least square means and SEM were presented, and statistical significance was set at *p* < 0.05.

## 3. Results 

### 3.1. Object Memory Test

**Figure 3:**
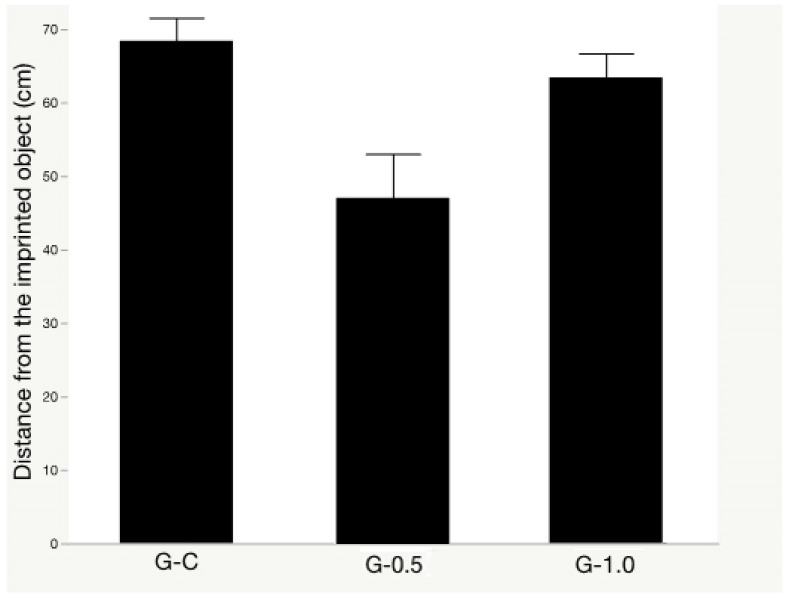
The synbiotic effects on object memory test of broilers exposed to acute heat stress. Treatments: a regular diet supplemented with 0 (G-C), 0.5 (G-0.5), and 1 (G-1.0) g kg^−1^ synbiotic, respectively. Data presented as means ± SE (*n* = 8, 16 birds/treatment).

The synbiotic effects on the object memory test are presented in Figure 3. The object memory test was not affected by the synbiotic supplementation regardless of its levels (*p* = 0.062).

### 3.2. Isolation Test

**Figure 4:**
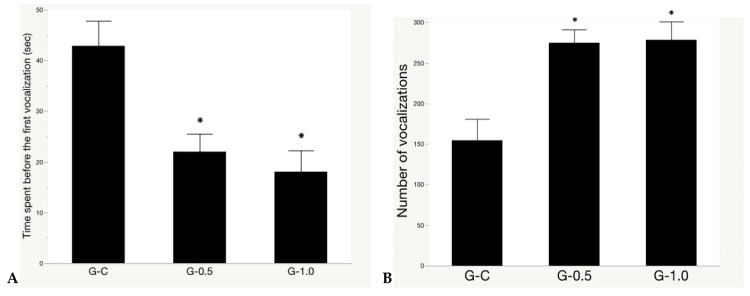
The synbiotic effect on isolation test of broilers exposed to chronic heat stress. (**A**) The latency to give the first vocalization; and (**B**) the number of vocalizations during the test. Treatments: a regular diet supplemented with 0 (G-C), 0.5 (G-0.5), and 1 (G-1.0) g kg^−1^ synbiotic, respectively. * significant difference (*p* < 0.05) from controls. Data presented as means ± SE (n = 8, 16 birds/treatment).

The synbiotic effects on the isolation test are presented in Figure 4. Synbiotic-fed birds had a shorter latency period before giving the first vocalization (*p* = 0.001. Figure 4A), with a higher number of vocalizations during the isolation test (*p* = 0.001. Figure 4B) regardless of the dosage.

### 3.3. Novel Object Test, Touch Test, and Tonic Immobility (TI)Test

The synbiotic effects on the novel object, touch, and TI tests are presented in Table 2. Compared to controls, the G-1.0 group had higher number of birds close to humans during the touch test (*p* = 0.001). However, there were no treatment effects on both the novel object and TI tests (*p* > 0.05).

### 3.4. Plasma Tryptophan and Corticosterone Analyses 

The synbiotic effects on plasma tryptophan and corticosterone concentration are presented in Table 3. Plasma levels of tryptophan were significantly elevated in the G-1.0 group compared to controls, while the levels of the G-0.5 group were intermediate (*p* = 0.043). However, plasma CORT concentrations were not affected by the dietary treatment, regardless of the concentration, in broiler chickens subjected to HS (*p* = 0.124).

### 3.5. Heterophil/Lymphocyte (H/L) Ratio

The synbiotic effects on Heterophil/Lymphocyte (H/L) ratio are presented in Table 3. In the current study, H/L ratios were reduced in synbiotic-fed broilers following HS with a dosage effect; G-1.0 had the highest decrease (*p* = 0.001).

## 4. Discussion

In the present study, the thermal condition of 32 °C daily for 9 h is guaranteed to cause HS according to previous studies [4,41,46,47,48,49]. Heat stress has a profound deleterious impact on broiler health and well-being. It suppresses immunity and disrupts physiological homeostasis, leading to hypersensitivity to neuroinflammation and metabolic disorders; consequently, production performance is impaired and diminished body weight, body weight gain, and increased feed conversion ratios are noted [50]. These alterations have a harmful effect on neurons and their function, causing brain ultrastructural damage and dysfunction, including mental and emotional disorders expressed at both memory and fear responsive levels [51,52]. The current findings indicated that the dietary synbiotic supplement improves the fear state and related stress response in broiler chickens under HS conditions. Interestingly, in one of our parallel studies, the body weight of the synbiotic-fed broilers at 42 day was remarkably improved as compared to controls following HS, regardless of synbiotic dosage [41]. 

Synbiotic administration has been used as a biotherapy for a variety of human diseases, including ageing- and Alzheimer’s-disease-associated memory loss and cognitive impairment [53,54,55]. In the present study, the object memory test was not affected by the synbiotic supplementation regardless of its level. Failure to observe any treatment effects could be associated with multiple factors, such as chicks’ age, the length of stimulation, the synbiotic concentration and the length of feeding time when the test was conducted. The current results indicate that the synbiotic under the current condition (Acute HS) may be not suitable for activating neuronal plasticity; however, previous results reported that birds’ ability to form a strong spatial memory is affected by early imprinting [17,56]. Similarly, Romo-Araiza et al. [57] reported that synbiotic supplementation (*E. faecium* + agave unulin, daily oral gavage for a 5-week period) improved the performance of Sprague-Dawley male rats in the spatial test (Morris water maze test), but not when associated with a memory test (Pavlovian autoshaping test). The improvement in spatial memory in rats was correlated with the reduced concentrations of proinflammatory cytokines (such as IL-1β) and increased levels of brain-derived neurotrophic factor (BDNF) in the hippocampus as well as the increased butyrate levels in feces. These findings provide clues for future investigations into the effect of synbiotic administration on memory ability, especially focusing on the alterations in butyrate, BDNF, and proinflammatory cytokine concentrations in the hippocampus, which have been associated with learning and memory processes as well as brain insults including HS. 

Modern commercial chickens, after generations of selection to increase traits directly related to production, have become increasingly sensitive to stress stimulations [58,59] and develop fear reactions when exposed to unpredictable, sudden or aversive stressors [60]. The fear test, (such as human touching test), isolation test (or open field test), and TI test, as well as the novel object test, are often used for measuring fearfulness in animals, including chickens [15,61]. Our findings suggest that dietary supplementation of the synbiotic can protect broilers from heightened fear. In the current study, compared to controls, synbiotic-fed groups had a higher number of birds that were close to humans during the touch test and a shorter latency period before the first vocalization, with a higher number of vocalizations during the isolation test. However, there were no treatment effects in both the novel object and TI tests. The latency of a bird to right itself during the TI test was 5.50 min (G-C), 4.07 min (G-0.5), and 3.55 min (G-1.0). Previously published results have reported a latency of bird to right itself in the TI test of 2 min for broilers reared in thermal-neutral conditions [62]. Similar to our results, Ghareeb et al. [62] reported no effect on TI test in broilers fed with a dietary supplement of *Lactobacillus sp.* for 5 weeks. In rodent studies, however, supplementation of *Lactobacillus rhamnosus* and *Lactobacillus helveticus* [63,64], *Bifidobacterium infantis* [65] restored the fear responses in maternal-separation-stressed infant rats by reversing the stress effects on neural circuits. Although the mechanisms underlying the dietary synbiotic supplement improving the fear responses of broilers were not examined in this study, they may be attributed to its functions in producing neuroactive substances affecting brain neural signaling via the enteric nervous system, the vagal afferents, and/or the bloodstream [66,67,68]. 

Among the neuroactive factors and their pathways, the central serotonergic system plays a key role in emotional coordination in humans and animals, which can be disrupted by heightened fear following a variety of stimulations [69] including HS [70]. The administration of tryptophan reduced the deleterious effects on the production performance of broiler chickens reared during the hot season in a tropical environment [71]. Several beneficial bacteria of synbiotics or probiotics have functions in the synthesis and release of serotonin through regulating tryptophan metabolism in the GIT [72,73]. In support of the hypothesis, the plasma concentrations of tryptophan were significantly elevated in the G-1.0 group compared to controls: 107.50 µmol/L (G-1.0) > 104.87 µmol/L (G-0.5) > 103.62 µmol/L (G-C). Previously published findings have reported a plasma tryptophan concentration of 107 µmol/L for broilers reared in normal environmental conditions [73]. Similar to the current findings, Clarke et al. [74] reported that the gut bacteria from the bedding and fecal matter directly regulate the plasma tryptophan concentrations in Swiss Webster mice. Tryptophan, but not serotonin, can pass the blood–brain barrier, and is an essential amino acid for serotonin synthesis. Tryptophan represents a critical component of serotonergic functions [75] participating in thermoregulation by reducing HS-linked oxidative stress [76], and acts directly as an important determinant of mood, fear, and related behaviors [77]. The synbiotic supplementation might exert an influence on tryptophan metabolism, resulting in an increase in brain serotonin concentration via the microbiome-gut–brain axis [78].

Numerous studies have evidenced that the HPA axis is involved in stress-induced fear response [12,79]. The release of CORT is correlated with the development of fearfulness in animals in response to various internal and external stimulations [80] via serial regulating pathways [81]. For example, fearfulness induces activation of the amygdala nucleus through the afferent neural circuits, which, in turn, activates hypothalamic neurons through both the stria terminalis bed nucleus and the brain stem raphe nucleus. Then, the corticotropic-releasing hormone (CRH) is released from the hypothalamic paraventricular nucleus, which, in turn, stimulates the release of adrenocorticotrophic hormone (ACTH) in the pituitary gland. ACTH promotes CORT secretion from the adrenal cortex into the blood stream [81].

Shi et al. [82] reported that hens kept in colony cages had higher plasma CORT with greater fearfulness and feather damage compared to hens housed in colony cages with nest boxes. In contrast, Peixoto et al. [83] reported that the effect of maternal stress on fearfulness in laying hens is not directly mediated by CORT. Similarly, in the current study, plasma CORT concentrations were not affected by the dietary treatment, regardless of its concentrations: 2.37 ng/mL (G-C), 2.19 ng/mL (G-0.5), 2.24 ng/mL (G-1.0). Previously published results have reported a plasma CORT concentration of 1.58 ng/mL for broilers reared in thermal-neutral conditions [84]. The absence of a treatment effect on the plasma CORT concentrations could be attributed to the characteristics of stressors and types of synbiotic or probiotic [85]. For example, Kridtayopas et al. [34] reported that a synbiotic-supplemented diet (mannanoligosaccharide mixed with *Bacillus subtilis* and *Bacillus licheniformis*) reduced the plasma CORT concentrations in broilers reared under a high stocking density, while Cengiz et al. [86] reported that dietary probiotic (*Lactobacillus acidophilus*, *Lactobacillus casei*, *Enterococcus faecium*, and *Bifidobacterium thermophilus*) did not affect CORT concentrations in broilers reared at different stocking densities (10 birds/m^2^ and 20 birds/m^2^). In addition, the hot ambient temperature elevated the HPA reactions in broilers [34] and may overcome the synbiotic effect on CORT synthesis and release from the adrenal glands. However, this hypothesis will be tested in future studies.

A variety of stressors, including HS, can suppress broiler chicken immunity by elevating the number of heterophils (neutrophils in mammals) while reducing the count of lymphocytes, leading to an increase in the H/L ratio. The H/L ratio has been widely used as a credible indicator of HS response in various animals, including broilers [87]. In the current study, H/L ratios were reduced in synbiotic-fed broilers following HS with a dosage effect, with the G-1.0 supporting the largest decrease: 0.79 (G-C) > 0.58 (G-0.5) > 0.43 (G-1.0). Previously published results have reported an H/L ratio of 0.50 for broilers at thermal-neutral conditions [88]. Similarly, decreased H/L ratios have been reported in probiotic (*bacillus subtilis)*-fed broilers under HS [48] and synbiotic (mannanoligosaccharide plus *bacillus subtilis*)-fed broilers under social stress [34]. The decreased H/L ratios in synbiotic-fed broilers may be attributed to regulation of the local and systemic immune organs by inhibiting pathogen colonization and related proinflammatory factors via the improvement in gut ecology [34].

The data suggest that the synbiotic-reduced HS response and related emotional disorder may mainly be induced through increasing the activation of the serotonergic system via the microbiota–gut–brain axis.

## 5. Conclusions

In the current study, the synbiotic supplement reduced broiler fear responses, as indicated by the outcomes of the touch test, and reduced the latency period before the first vocalization, with a higher frequency of vocalizations reported during the isolation test. In addition, the synbiotic effects on fearfulness were correlated with reduced H/L ratios and elevated plasma tryptophan, particularly in the G-1.0 group. Overall, our results indicate that dietary synbiotic supplementation may be a beneficial tool for ameliorating HS deleterious effects on broilers reared during summer hot seasons.

## Figures and Tables

**Table 1:** Components of base diet ^1^, separated by the growth phase ^2^.

Ingredient %	Starter (1–14 day)	Grower (15–28 day)	Finisher (29–42 day)
Corn ground	57.66	63.76	66.9
Soybean meal 47.5%	35.27	29.68	26.3
Soybean oil degummed	3	3	3.52
Calcium carbonate	1.41	1.38	1.49
Phosphate monocalcium	1.42	1.02	0.82
L-Lysine	0.11	0.1	0.02
Salt plain	0.48	0.46	0.48
L-Threonine 98%	0.06	0.04	0
DL-Methionine	0.24	0.21	0.12
Poultry turkey starter	0.35	0.35	0.35
Calculated Analysis ^3^			
Crude protein %	23.4	22.8	19.2
Poultry ME kcal/kg	3050	3151	3200
Calcium %	0.95	0.85	0.75
Available phosphorus %	0.50	0.44	0.36
Methionine %	0.66	0.59	0.53
Methionine+Cystine %	1.04	0.97	0.86
Lysine %	1.42	1.29	1.09
Threonine %	0.97	0.89	0.74
Na %	0.22	0.20	0.19

^1^ The ration formulation was produced according to Aviagen [40], and the treatments were the regular diets supplemented with 0 (G-C), 0.5 (G-0.5), and 1 (G-1.0) g kg^−1^synbiotic, respectively. ^2^ The diets were formulated by the Purdue University Feed Mill. (w. Lafayette, IN, USA). ^3^ Provided per kilogram of diet: vitamin A, 13.233 IU; vitamin D3, 6.636 IU; vitamin E, 44.1 IU; vitamin K, 4.5 mg; thiamine, 2.21 mg; riboflavin, 6.6 mg; pantothenic acid, 24.3 mg; niacin, 88.2 mg; pyridoxine, 3.31 mg; folic acid, 1.10 mg; biotin, 0.33 mg; vitamin B12, 24.8 μg; choline, 669.8 mg; iron from ferrous sulfate, 50.1 mg; copper from copper sulfate, 7.7 mg; manganese from manganese oxide, 125.1 mg; zinc from zinc oxide, 125.1 mg; iodine from ethylene diamine dihydroidide, 2.10 mg; selenium from sodium selenite, 0.30 mg.

**Table 2:** Effect of the dietary synbiotic supplementation on the novel object, touch, and tonic immobility tests of heat-stressed broiler chickens.

Treatments ^1^	G-C	G-0.5	G-1.0	SEM	*p*-Value
Novel object test ^2^					
Number of birds within 30 cm from the object	25.87	22.50	25.50	2.67	0.627
Touch test ^2^					
Number of touched birds	25.12 ^b^	33.62 ^b^	78.87 ^a^	4.83	0.001
Tonic immobility test ^3^					
Latency of bird to right itself (min)	5.50	4.07	3.55	0.74	0.185

^a,b^ Means ± SEM with different superscripts in the same row differ significantly (*p* < 0.05. *n* = 8). ^1^ A regular diet supplemented with 0 (G-C), 0.5 (G-0.5), and 1 (G-1.0) g kg^−1^ synbiotic, respectively. ^2^ Data were collected from 120 birds/treatment. ^3^ Data were collected from 16 birds/treatment.

**Table 3:** Effect of dietary synbiotic supplementation on Heterophil/Lymphocyte ratio, plasma concentrations of corticosterone and tryptophan of the heat-stressed broiler chickens from 15 to 42 day.

Treatments ^1^	G-C	G-0.5	G-1.0	SEM	*p*-Value
H/L ratio	0.79 ^a^	0.58 ^b^	0.43 ^c^	0.02	0.001
Corticosterone (ng/mL)	2.37	2.19	2.24	0.06	0.124
Tryptophan (µmol/L)	103.62 ^b^	104.87 ^ab^	107.50 ^a^	1.03	0.043

^a,b,c^ Means ± SEM with different superscripts in the same row differ significantly (*p* < 0.05. n = 8 from 16 birds per treatment). ^1^ A regular diet supplemented with 0 (G-C), 0.5 (G-0.5), and 1 (G-1.0) g kg^−1^ synbiotic, respectively.
